# Monte Carlo study of noise-induced phase angle errors in low-frequency tetrapolar bioimpedance measurements

**DOI:** 10.2478/joeb-2026-0017

**Published:** 2026-11-04

**Authors:** Jie Hou, Sisay Mebre Abie, Ørjan G. Martinsen

**Affiliations:** Department of Physics, University of Oslo, Oslo, Norway; Department of Clinical and Biomedical Engineering, Oslo University Hospital, Oslo, Norway

**Keywords:** Bioimpedance, phase angle, tetrapolar, lock-in detection, Monte Carlo simulation, signal-to-noise ratio

## Abstract

Phase angles measured at low frequencies with the tetrapolar technique are sometimes scattered or even positive. We used Monte Carlo simulations of lock-in detection to quantify how large phase errors that measurement noise alone can cause. For a signal-to-noise ratio after demodulation (SNR_out_) of about 3 or higher, the standard deviation of the phase error was close to 57° divided by SNR_out_, and 95% of the errors were within about twice this value. At SNR_out_ = 10 the error standard deviation was 6°; at SNR_out_ = 1 it was 64° and 16% of the measurements were wrong by more than 90°. As SNR_out_ decreased towards zero, the phase gradually approached a completely random value. In an illustrative low-frequency tetrapolar measurement, the absolute phase errors were small (standard deviation 0.007° at 1 Hz and 0.04° at 0.1 Hz), but the true tissue phase was even smaller. The error was therefore 1.2 times the true phase at 1 Hz and 37 times at 0.1 Hz, and the measured phase had the wrong sign in 20% and 49% of the measurements, respectively. The sign of a small phase angle is only reliable (wrong in less than 1% of measurements) when the true phase is larger than 2.3 times the error standard deviation.

## Introduction

The phase angle is a widely used bioimpedance parameter [1]. Tetrapolar measurements greatly reduce the influence of the electrode polarization impedance [2], but may give positive phase angles. A positive transfer-impedance phase can arise from the current paths and sensitivity distribution of the electrode configuration [3]. Random measurement noise is another possible cause.

At low frequencies two effects combine. The phase angle of tissue becomes small, and the signal-to-noise ratio (SNR) decreases, because the polarization impedance of the current-carrying electrodes limits the injected current, while electrode noise [4] and amplifier 1/*f* noise increase.

The aim of this communication is to show how large the phase error caused by noise can be in lock-in based measurements [5, 6], both in degrees and relative to the true phase, and when it makes the sign of the phase unreliable. The statistics of the phase of a sinusoid in Gaussian noise are well established [7]. Our contribution is their application to low-frequency tetrapolar measurements, showing that the phase sign can be unreliable even at a very high SNR when the tissue phase is small.

## Materials and methods

### Lock-in detection

The measured voltage was a sinusoid with peak amplitude *A*_sig_, frequency *f*_sig_ and true phase *ϕ*, with additive Gaussian noise. It was multiplied by a sine and a cosine reference at *f*_sig_ and averaged over a measurement time *T* of an integer number of cycles, giving the in-phase and quadrature outputs *X* and *Y*. The measured phase was

(1) $$\phi_{\text{meas}}=\text{atan}2(Y,X).$$

Without noise, (*X, Y* ) is a point at distance *M*_0_ = *A*_sig_/2 from the origin (unit reference amplitude) at angle *ϕ*. Noise spreads this point into a cloud, and the phase error is the angle between the noisy and the true point.

### Monte Carlo model and simple error estimates

In the Monte Carlo model, Gaussian noise with standard deviation *σ_X_* was added directly to *X* and *Y*. We define the SNR after demodulation as SNR_out_ = *M*_0_/*σ_X_*. This is an amplitude ratio; the corresponding power ratio is ${\text{SNR}}_{\text{out}}^{2}$ , or 20 log_10_ SNR_out_ in dB. For example, SNR_out_ = 0.5 (−6 dB) means that the noise standard deviation in *X* and *Y* is twice the signal amplitude after demodulation. Averaging reduces the noise, and

(2) $${\text{SNR}}_{\text{out}}=A_{\text{sig}}\sqrt{T/S_{n}},$$

where *S_n_* is the one-sided noise power spectral density (V^2^/Hz) at *f*_sig_. For white noise with standard deviation *σ_n_* and *N_s_* samples, ${\text{SNR}}_{\text{out}}={\text{SNR}}_{\text{in}}\sqrt{N_{s}}$ , with ${\text{SNR}}_{\text{in}}=A_{\text{sig}}/(\sqrt{2}\sigma_{n})$ (rms signal to rms noise, also an amplitude ratio).

When the noise is small compared with *M*_0_ (SNR_out_ ≳ 3), the standard deviation of the phase error is

(3) $$\sigma_{\phi }\approx \frac{57.3{}^{\circ}}{{\text{SNR}}_{\text{out}}},$$

and about 95% of the errors lie within ±2*σ_ϕ_*. The measured phase gets the wrong sign when the noise moves the point across the *X* axis. For −180° *< ϕ <* 0, this happens exactly when *Y >* 0, with probability

(4) $$P_{\text{sign}}=\Phi (-{\text{SNR}}_{\text{out}}|\sin\phi |),$$

where Φ is the standard normal cumulative distribution function. For a small true phase, SNR_out_| sin *ϕ| ≈ |ϕ|/σ_ϕ_*, so the sign is wrong in less than 1% of the measurements when

(5) $$|\phi |>2.3\;\sigma_{\phi }.$$

The phase error was defined as *ϕ*_meas_ *−ϕ*, wrapped to the range (−180°, 180°].

### Simulations with white noise

The Monte Carlo model was first compared with a full time-domain simulation, in which a new noise sequence was added to the sampled signal before mixing and averaging. The reference case was a 10 Hz signal with *A*_sig_ = 0.05 and *ϕ* = −40°, white noise with *σ_n_* = 0.6 (SNR_in_ = 0.059, i.e. −24.6 dB, with a noise power about 290 times the signal power), sampling at 10 kHz and 10 cycles (*T* = 1 s, *N_s_* = 10 000). The number of cycles, the frequency (with 10 cycles) and *σ_n_* were then varied. Finally, the phase error was calculated for SNR_out_ from 0.05 to 100. Each case used 10 000–200 000 simulated measurements.

### Low-frequency tetrapolar measurement

The tissue was described by a Cole model [1], *Z_t_* = *R_∞_* + (*R*_0_ *−R_∞_*)/[1 +(*jf/f_c_* )*^α^*], with *R*_0_ = 100 Ω, *R_∞_* = 60 Ω, *f_c_* = 50 kHz and *α* = 0.75, used as both the load and the transfer impedance. Each electrode had the impedance *Z_e_* = 100 Ω+ 500 kΩ *·* (*j f*/1 Hz)^−0.8^, representing a high-impedance skin contact. The current source delivered at most 100 *µ*A, limited by a compliance voltage of 2.5 V over 2*Z_e_* + *Z_t_*. At the limit, the current was assumed to remain sinusoidal with a reduced amplitude. Below about 100 Hz the current was set by this voltage, *I* = 2.5 V/|2*Z_e_* + *Z_t_*|, which is the same as for an instrument that applies a constant voltage and measures the current. The low-frequency results therefore also apply to voltage-driven instruments, except that the noise of their current measurement adds to the phase error and was not included here. The pick-up voltage was the current times |*Z_t_*|, and the true phase was the phase of *Z_t_*, assuming that the lock-in reference represents the actual injected current. The noise was the thermal noise of the two pick-up electrodes, 8*k_B_T*_K_Re{*Z_e_*} with *T*_K_ = 310 K, plus amplifier noise with the power spectral density $e_{n}^{2}(1+f_{1/f}/f)$ , where $e_{n}=10\;\text{nV}/\sqrt{\text{Hz}}$ and *f*_1_*_/f_* = 50 Hz. Real electrode noise can be considerably larger [4]. The parameters are illustrative.

The frequency was swept from 0.1 Hz to 100 kHz with 10 or 100 cycles and 20 000 simulated measurements per frequency. As a baseline, the sweep was repeated with constant current and white noise only. The electrode impedance, the electrode noise power, the amplifier noise, the compliance voltage and an additional low-frequency tissue dispersion ∆*R*/[1 + (*jf*/10 Hz)^0.7^] with ∆*R* = 1 or 10 Ω, added to *Z_t_*, were varied one at a time, using Eq. (4) on a frequency grid from 0.01 to 1000 Hz. The Monte Carlo model was also checked against time-domain simulations with noise generated with the same spectrum at 0.1, 1 and 10 Hz. All simulations were done in Python with NumPy and SciPy, with a fixed random seed.

### Ethical approval

The conducted research is not related to either human or animal use.

## Results

### Phase error and SNR

In the reference case (SNR_out_ = 5.9), the time-domain simulation and the Monte Carlo model gave the same (*X, Y* ) cloud and phase distribution (Fig. 1b,c). The mean phase was −39.9° and −40.0°, and the error standard deviation 9.8° and 9.9°, compared with 9.7° from Eq. (3). Single measurements deviated by more than 20°, even though the noise was averaged over 10 000 samples.

**Figure 1:**
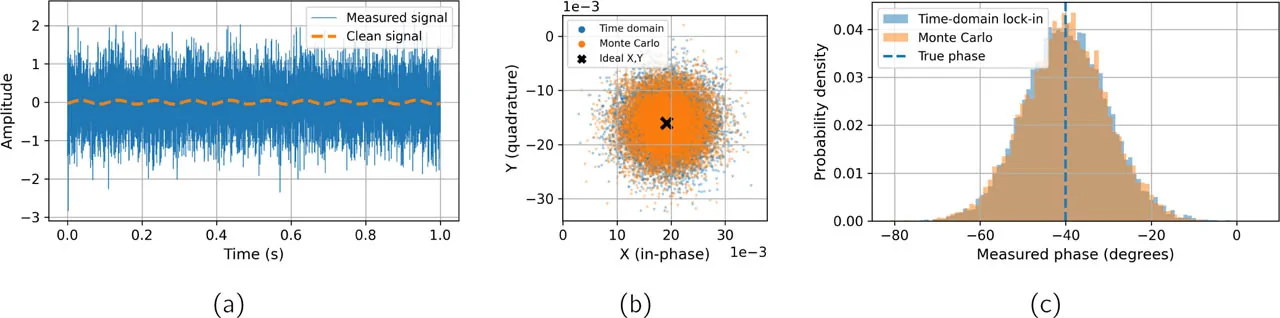
White-noise reference case. (a) 10 Hz signal (*A*_sig_ = 0.05) with noise (*σ_n_* = 0.6); the clean signal is dashed. (b) Lock-in outputs (*X, Y* ) and (c) measured phase from the time-domain simulation and the Monte Carlo model; the cross marks the noise-free point and the dashed line the true phase.

Table 1 shows how large the error can be. For SNR_out_ *≥* 3, the error standard deviation agreed with Eq. (3) within 10%, and 95% of the errors were within ±2*σ_ϕ_*. At lower SNR, Eq. (3) first underestimated and then overestimated the error, since the error standard deviation cannot exceed the 104° of a completely random phase. Below SNR_out_ *≈* 2, errors larger than 90°, i.e. a phase in the wrong half-plane, appeared. As SNR_out_ decreased further, the error distribution gradually approached that of a completely random phase. At SNR_out_ = 0.5 the measured phase was very uncertain but still informative (standard deviation 84°, 31% of errors larger than 90°), whereas at 0.1 and 0.05 it was nearly random. The error decreased with the square root of the number of cycles (Fig. 2a), increased with frequency when the number of cycles was fixed, because the measurement became shorter (Fig. 2b), and was proportional to the noise level (Fig. 2c).

**Figure 2:**
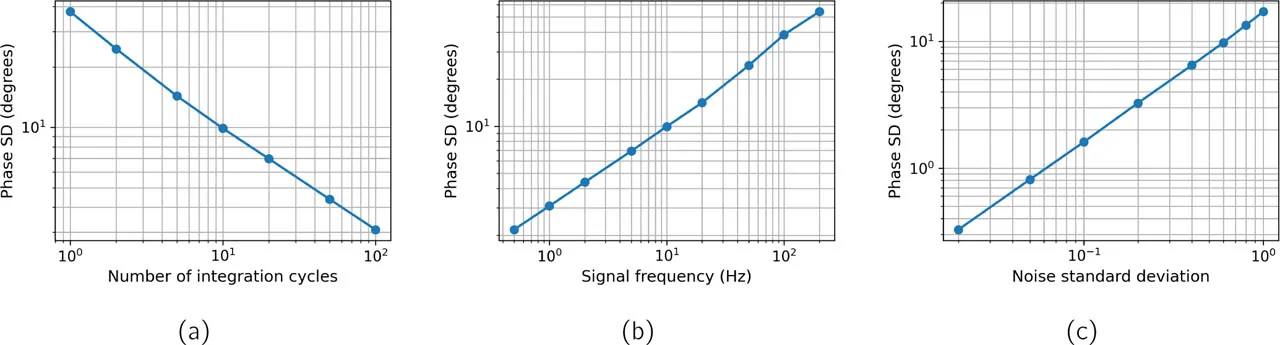
White-noise case: standard deviation of the phase error as a function of (a) the number of integration cycles at 10 Hz, (b) the signal frequency with 10 cycles, and (c) the noise standard deviation at 10 Hz and 10 cycles.

**Table 1:** Phase error versus SNR after demodulation (amplitude ratio; Monte Carlo, 200 000 simulated measurements per row). The last row is the limit *SNR_out_ →* 0, i.e. a completely random phase.

SNR_out_	Error SD (deg)	95% of errors within (deg)	Errors *>* 90° (%)
0.05	102	±170	48
0.1	100	±170	46
0.25	94	±168	40
0.5	84	±162	31
1	64	±140	16
2	35	±69	2.3
3	21	±41	0.1
5	12	±23	0
10	5.8	±11	0
20	2.9	±5.6	0
100	0.57	±1.1	0

→ 0 (random)	104	±171	50

### Low-frequency tetrapolar measurement

The Monte Carlo model agreed with the time-domain simulations with coloured noise within 4%. Above about 100 Hz the current was 100 *µ*A and the pick-up voltage about 10 mV (Fig. 3a). Towards low frequencies, the compliance limit reduced the current to 2.5 *µ*A at 1 Hz and 0.4 *µ*A at 0.1 Hz, and the noise increased. With 10 cycles, SNR_out_ fell from 1.9 × 10^5^ at 100 Hz to 7800 at 1 Hz and 1400 at 0.1 Hz. With constant current and white noise, in contrast, the error continued to decrease towards low frequencies (Fig. 3b).

**Figure 3:**
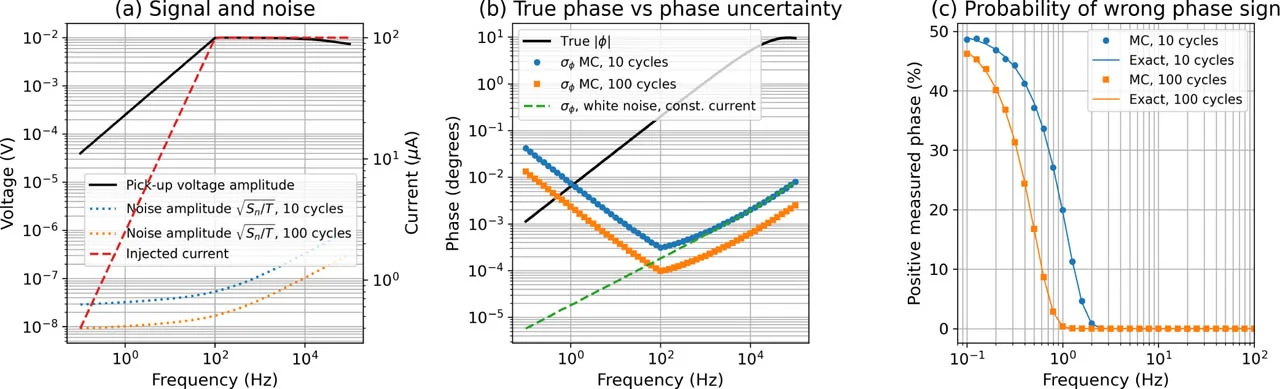
Low-frequency tetrapolar measurement. (a) Pick-up voltage, noise amplitude after averaging over 10 and 100 cycles (left axis) and injected current (right axis). (b) True phase magnitude and standard deviation of the phase error for 10 and 100 cycles (markers: Monte Carlo; lines: Eq. (3)), and for constant current with white noise. (c) Fraction of measurements with a positive (wrong-sign) phase (markers: Monte Carlo; lines: Eq. (4)).

Because SNR_out_ remained high, the absolute errors were small: the error standard deviation was 0.0003° at 100 Hz, 0.0074° at 1 Hz and 0.042° at 0.1 Hz, so 95% of the errors were within ±0.015° and ±0.08° at 1 and 0.1 Hz. However, the true phase fell from −9.5° at 100 kHz to −0.0063° at 1 Hz and −0.0011° at 0.1 Hz. Relative to the true phase, the error standard deviation was 4% at 10 Hz, 120% at 1 Hz, 330% at 0.5 Hz and 3700% at 0.1 Hz. With 10 cycles, the measured phase was positive in 0.8% of the measurements at 2 Hz, 20% at 1 Hz, 38% at 0.5 Hz and 49% at 0.1 Hz (Fig. 3c). With 100 cycles, the corresponding values at 1, 0.5 and 0.1 Hz were 0.3%, 17% and 47%.

In the sensitivity analysis (Table 2), the frequency below which sign errors exceeded 1% ranged from 0.06 to 8.7 Hz. An additional tissue dispersion of only 1 Ω increased the true phase at 1 Hz fourteen-fold and reduced the sign errors at 1 Hz below 0.1%.

**Table 2:** Sensitivity analysis (10 cycles): true phase at 1 Hz, percentage of positive phases at 1 and 0.1 Hz, and the highest frequency *f*_1%_ where this exceeds 1%.

Case	*ϕ*_1 Hz_ (deg)	*P*_1 Hz_ (%)	*P*_0.1 Hz_ (%)	*f*_1%_ (Hz)
Reference	−0.006	20	49	2.0
|*Z_e_* (1 Hz)| = 100 kΩ	−0.006	< 0.1	44	0.56
|*Z_e_* (1 Hz)| = 2.5 MΩ	−0.006	46	50	8.7
Electrode noise power ×10	−0.006	36	50	3.6
Amp. 3 nV, corner 5 Hz	−0.006	12	48	1.6
Amp. 30 nV	−0.006	35	50	3.3
Compliance 10 V	−0.006	< 0.1	46	0.77
Dispersion 1 Ω	−0.089	< 0.1	31	0.30
Dispersion 10 Ω	−0.77	< 0.1	< 0.1	0.06

## Discussion

The simulations show two ways in which noise causes large phase errors. When SNR_out_ is low, the absolute error is large: about 57°/SNR_out_ for SNR_out_ ≳ 3, i.e. more than 10° for SNR_out_ < 5, with occasional errors above 90° for SNR_out_ < 2 (Table 1). When SNR_out_ is high but the true phase is very small, as in the low-frequency tetrapolar example, the absolute error is only hundredths of a degree, but it can still be larger than the phase itself. The sign is then unreliable, and Eq. (5) shows that a reliable sign requires the true phase to exceed 2.3 times the error standard deviation.

In our tetrapolar example only thermal electrode noise and a low-noise amplifier were included, so the first (low-SNR) case did not occur. Large absolute errors require much more noise: with 10 cycles, SNR_out_ = 5 (error SD 12°) would require a noise density of about 160 $\mu \text{V}/\sqrt{\text{Hz}}$ at 1 Hz and 80 $\mu \text{V}/\sqrt{\text{Hz}}$ at 0.1 Hz, i.e. about 1500 and 270 times the modelled values. Such noise levels may occur with poor or dry electrodes, motion or interference. To predict the phase error of a given set-up, the measured noise spectrum of the instrument should be used in Eq. (2).

Eq. (3) gives a simple way to estimate the expected phase error of a measurement from its SNR. Since the true phase is unknown in experiments, the error should be estimated from the measured noise or from repeated measurements. The error decreases only with the square root of the measurement time: keeping sign errors below 1% required 74 cycles at 1 Hz, 583 at 0.5 Hz and 7.5 × 10^4^ (about nine days) at 0.1 Hz. Lower electrode impedance or a higher compliance voltage were more effective (Table 2). A low frequency by itself does not give larger errors (Fig. 2b); in the tetrapolar model the SNR falls because of the current limitation and the increasing noise.

Noise causes scatter around the true phase, not a systematic positive shift, and the fraction of positive phases stays below 50%. The absolute errors in our tetrapolar example do not explain positive phases of several degrees, which point to other mechanisms such as the sensitivity distribution of the electrode configuration [3]. The results depend strongly on the low-frequency tissue phase, and a single extrapolated Cole dispersion illustrates a near-zero-phase case. Other limitations are the thermal-only electrode noise, the simplified circuit, the noise in the current measurement of voltage-driven instruments, and the neglect of mains interference, drift and practical lock-in filters [6]. A bench test with a known impedance and controlled noise would be a natural next step.

## Conclusion

For SNR_out_ ≳ 3, the standard deviation of the noise-induced phase error is about 57°/SNR_out_, with 95% of the errors within twice this value. At low SNR the error reaches tens of degrees and can exceed 90°. At high SNR the error is small in degrees, but in low-frequency tetrapolar measurements, where the true phase approaches zero, it can exceed the phase itself and give the wrong sign in up to half of the measurements. The sign of a small phase is reliable only when the true phase is larger than 2.3 times the error standard deviation.
